# Improved Robust Model Predictive Trajectory Tracking Control for Intelligent Vehicles Based on Multi-Cell Hyperbody Vertex Modeling and Double-Layer Optimization

**DOI:** 10.3390/s25216537

**Published:** 2025-10-23

**Authors:** Xiaoyu Wang, Guowei Dou, Te Chen, Jiankang Lu

**Affiliations:** 1 School of Mechanical and Electrical Engineering, Suzhou Polytechnic University, Suzhou 215000, China; 2 Automotive Engineering Research Institute, Jiangsu University, Zhenjiang 212013, China; 3 Robotics and Intelligent Equipment Engineering Research Center of Jiangsu Province, Suzhou Polytechnic University, Suzhou 215000, China

**Keywords:** vehicle sideslip angle, fusion estimation, distributed drive electric vehicle, uncertainty, tire nonlinearity

## Abstract

Aiming at the problem of model parameter perturbation in vehicle trajectory tracking control, an improved robust model predictive control (RMPC) method is proposed. Based on the two-degree-of-freedom vehicle model and Serret Frenet error model, a multi-cell hypercube vertex modeling is adopted to map the disturbance range of parameters such as vehicle speed and lateral stiffness to a set of vertices, and dynamic linear combination is achieved through normalized weights. The algorithm design mainly focuses on the dual-layer optimization of the switching mechanism, decomposing the infinite time domain problem into finite time domain optimization and terminal constraints. At the same time, it dynamically updates the vertex parameters to match time-varying uncertainties and then combines Lyapunov theory to design a control invariant set. The results show that in complex road conditions and vehicle state transitions, RMPC can reduce the peak lateral deviation from 1.0 m to 0.2 m, converge the heading deviation to within 2 deg, and significantly reduce the mean and root mean square values of control errors compared to traditional MPC, under the influence of vehicle model parameter perturbations. RMPC has good robustness and real-time performance.

## 1. Introduction

In contemporary intelligent vehicle systems, trajectory tracking control has emerged as a cornerstone technology for autonomous and semi-autonomous driving paradigms. As the core component of motion control in intelligent driving vehicles, trajectory tracking control performance directly determines operational safety margins and ride quality parameters [1,2,3]. Model accuracy is one of the important factors determining the effectiveness of vehicle trajectory tracking and stability control, while parameter uncertainty is a major challenge in the field of vehicle dynamics control, directly affecting model accuracy, trajectory tracking performance, and stability control effectiveness. The uncertainty of model parameters can lead to deviations between the actual motion state of the vehicle and the expected motion state of the controller [4,5,6]. Model-based trajectory-tracking controllers rely on nominal values of vehicle velocity and tire cornering stiffness. Whenever the true parameters deviate from these nominal values, the computed steering angle and traction force fail to match the ideal feedforward term, which immediately degrades lateral path tracking accuracy [7,8]. When there is uncertainty in the model parameters, it may disrupt the design conditions of the controller and compromise the stability of the closed-loop control system [9,10]. In some controllers designed based on linearized models, if the actual parameters of the vehicle exceed the approximate range of the linearized model, the nonlinear characteristics of the system will become significant, which may cause the controller to be unable to effectively suppress the divergence of the vehicle state, resulting in unstable phenomena during the driving process and threatening the safety of the vehicle [11,12].

In the existing literature, numerous scholars have proposed various trajectory tracking control strategies, such as traditional proportional integral derivative (PID) control. Although simple and feasible, its control accuracy and adaptability are significantly insufficient for complex dynamic systems and variable operating conditions of intelligent vehicles [13,14,15]. Sliding mode control guarantees robustness to matched disturbances, but the high-frequency switching excites chassis vibrations and shortens actuator life. Pure pursuit laws, derived from the kinematic bicycle model, only require a look-ahead distance, but they ignore dynamic forces; consequently, the preview gain that works for low-speed parking fails at highway speeds or on low-friction surfaces [16,17,18,19]. However, such methods often meet the high-performance and dynamic control requirements of vehicles when dealing with high-speed driving, complex road conditions, and changes in vehicle dynamic characteristics. In addition, the uncertainty of these parameters makes it difficult for control laws designed based on precise mathematical models to continue to function. Even if ideal trajectory tracking can be achieved at the initial moment, control errors will gradually accumulate or even disperse over time and changes in vehicle status. In severe cases, it may cause vehicle driving stability problems, greatly limiting the reliability and practicality of trajectory tracking control systems [20,21,22].

The model predictive control (MPC) algorithm has shown great potential in the field of intelligent vehicle trajectory tracking due to its unique rolling optimization, real-time feedback correction, and explicit constraint processing capabilities [23,24,25]. At every sampling instant, the optimizer computes the sequence of future steering angles and traction forces that minimizes the deviation from the reference path; the first element is applied, and the procedure is repeated, yielding a receding-horizon feedback policy [26,27,28]. Many studies have demonstrated the advantages of MPC in handling nonlinear dynamic characteristics of vehicles and multi-input multi-output coupling relationships, especially in some standardized driving scenarios such as lane keeping and cornering. Trajectory tracking control based on MPC can achieve good control effects. Some scholars have further improved its performance by improving the optimization algorithm of MPC, adjusting parameters such as prediction time domain and control time domain [29,30,31].

However, when accounting for model parameter uncertainties, traditional MPC algorithms will expose notable limitations. Owing to the high dependence of its control law generation on the precise description of the vehicle model, when there is a deviation in the model parameters, the gap between the predicted results and the actual vehicle motion state will gradually widen, leading to the accumulation of control input deviation and affecting the accuracy and stability of trajectory tracking [32,33,34,35]. To address these limitations, RMPC has been developed. Building upon traditional MPC, RMPC integrates robust control theory and uses methods such as conservative uncertainty set description, adding robust penalty terms to the optimization objective function, and designing feedback-based compensation mechanisms to compensate and correct model parameter uncertainty. In uncertain environments, RMPC ensures stable tracking of the reference trajectory while adhering to vehicle dynamics constraints and safety requirements. It provides strong theoretical and technical support for intelligent vehicles to achieve reliable and efficient trajectory tracking control in complex and changing real traffic scenarios, and has very important research significance [36,37]. It not only enhances the autonomous driving performance of intelligent vehicles but also plays a pivotal role in advancing intelligent transportation systems, enhancing road traffic efficiency and safety. It is one of the important directions in the current research field of intelligent vehicle trajectory tracking control.

With the rapid development of intelligent driving technology, vehicle trajectory tracking control faces severe challenges brought by model parameter uncertainty in complex working conditions. This manuscript systematically constructs a comprehensive vehicle model considering multi-cell uncertainty to address key issues such as parameter perturbations, nonlinear tire characteristics, and environmental disturbances in vehicle dynamics models. An RMPC algorithm based on improved LMI optimization is designed to improve vehicle trajectory tracking accuracy and dynamic stability. A multi-cell uncertainty modeling was conducted by combining a vehicle dynamics model and a trajectory tracking error model. By separating nominal parameters and disturbance terms, a hypercube vertex model covering multidimensional parameter perturbations such as vehicle speed and lateral stiffness was constructed, providing an accurate parameterized representation for robust control. We designed an improved LMI optimization strategy and proposed a dual-layer optimization framework based on a switching mechanism, which transforms the infinite time domain problem into a combination optimization of finite time domain and terminal boundary conditions, significantly reducing computational complexity. We have established a dynamic weight and real-time vertex update mechanism, which enhances the adaptability to time-varying uncertainty and alleviates the problem of vehicle model mismatch through dynamic adjustment of normalized weight coefficients and real-time reconstruction of vertex parameters.

The research contributions of this work are as follows. (1) A multi-cell model was constructed to describe the uncertainties of parameters such as vehicle speed and lateral stiffness. By adopting a dynamic weight combination approach, time-varying parameter adaptive modeling was achieved, and the infinite time domain robust control problem was transformed into a finite time domain optimization problem. (2) The Lyapunov–Krasovskii function was introduced to construct robust constraints, and the separation objective function minimization and maximum tolerance boundary solution were achieved based on the double-layer optimization structure. (3) A mechanism for dynamic adjustment of normalized weight coefficients and real-time vertex reconstruction was proposed to track and compensate for model parameter perturbations online, effectively alleviating the model mismatch problem and taking into account both control accuracy and real-time performance.

The rest of this paper is organized as follows. The modeling of the vehicle is presented in Section 2. The robust control method for trajectory tracking with parameter uncertainty is provided in Section 3. The simulation results are shown in Section 4. The experimental results are presented in Section 5, and the conclusive remarks are presented in Section 6.

## 2. Modeling

### 2.1. Vehicle Dynamic Model

**Figure 1:**
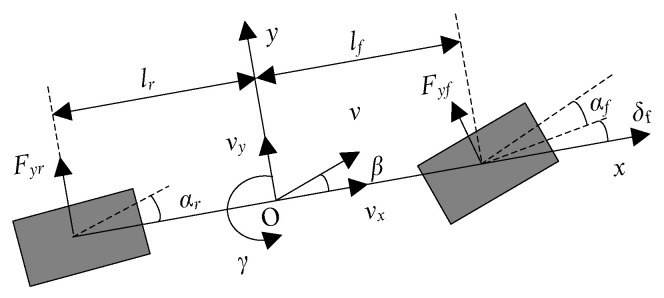
Vehicle dynamic model.

The vehicle dynamics equations with lateral and yaw degrees of freedom are established to characterize the dynamic mapping relationship between vehicle parameters. As shown in Figure 1, a dynamic coordinate system *xoy* is established, where the origin of the coordinate system coincides with the vehicle’s center of mass, the *x*-axis represents the longitudinal driving direction of the vehicle with the forward direction as positive, and the *y*-axis represents the lateral movement direction of the vehicle with the direction to the left of the forward direction as positive. The vehicle suspension system, as well as the pitching, rolling, and vertical movements of the vehicle, are ignored, and only the dynamic characteristics of the vehicle in the *xoy* plane are discussed. Meanwhile, all tire characteristics are considered to be consistent. Then, the dynamics equations of the vehicle’s single-track model can be expressed as

(1) $$m\left({\dot{v}}_{y}+v_{x}\gamma \right)=F_{yf}+F_{yr},$$

(2) $$I_{z}\dot{\gamma }=l_{f}F_{yf}-l_{r}F_{yr}+\Delta M_{z},$$

where *v_x_* represents the longitudinal vehicle speed, *v_y_* represents the lateral vehicle speed, *γ* represents the vehicle’s yaw rate, *m* represents the vehicle’s mass, *I_z_* represents the vehicle’s moment of inertia, *l_f_* and *l_r_* are the distances from the vehicle’s center of mass to the front and rear axles, respectively, *F_yf_* and F*_yr_* are the lateral forces of the front and rear axle tires, respectively, and $\Delta M_{z}$ are the additional yaw moments. When considering that the tire force is in the linear region, the calculation formulas of *F_yf_* and F*_yr_* can be expressed as

(3) $$\begin{array}{l} F_{yf}=-2C_{f}\alpha_{f}\\ F_{yr}=-2C_{r}\alpha_{r} \end{array},$$

where *C_f_* and *C_r_* represent the roll stiffness of the front and rear axle tires, respectively, and *α_f_* and *α_r_* represent the roll angles of the front and rear axle tires, respectively. The tire sideslip angle can be expressed as

(4) $$\begin{array}{l} \alpha_{f}=\delta_{f}-\left(l_{f}\gamma +v_{y}\right)/v_{x}\\ \alpha_{r}=\left(l_{r}\gamma -v_{y}\right)/v_{x} \end{array},$$

where *δ_f_* represents the steering angle of the front wheels. Combining Equations (1)–(4), the two-degree-of-freedom vehicle dynamics model can be obtained as

(5) $${\dot{v}}_{y}=-\frac{C_{f}+C_{r}}{mv_{x}}v_{y}-\left(v_{x}+\frac{C_{f}l_{f}-C_{r}l_{r}}{mv_{x}}\right)\gamma +\frac{C_{f}}{m}\delta_{f},$$

(6) $$\dot{\gamma }=-\frac{C_{f}l_{f}-C_{r}l_{r}}{I_{z}}\beta -\frac{C_{f}l_{f}^{2}+C_{r}l_{r}^{2}}{I_{z}v_{x}}\gamma +\frac{C_{f}l_{f}}{I_{z}}\delta_{f}+\frac{1}{I_{z}}\Delta M_{z},$$

### 2.2. Trajectory Tracking Control Error Model

When constructing the vehicle trajectory tracking control error model, this study adopts multi-dimensional deviation indicators to quantitatively evaluate the tracking performance. Among them, the lateral position deviation *e* is defined as the vertical distance between the vehicle’s center of mass in the *Y*-axis direction and the intended trajectory processed by the presight point. The heading angle error *ψ* characterizes the angular deviation between the current heading of the vehicle and the tangent direction of the ideal trajectory. It is worth noting that the selection of the presight distance *D_p_* needs to comprehensively consider the kinematic characteristics of the vehicle and the real-time control requirements, and can be dynamically calculated by the product of the vehicle speed and the presight time.

**Figure 2:**
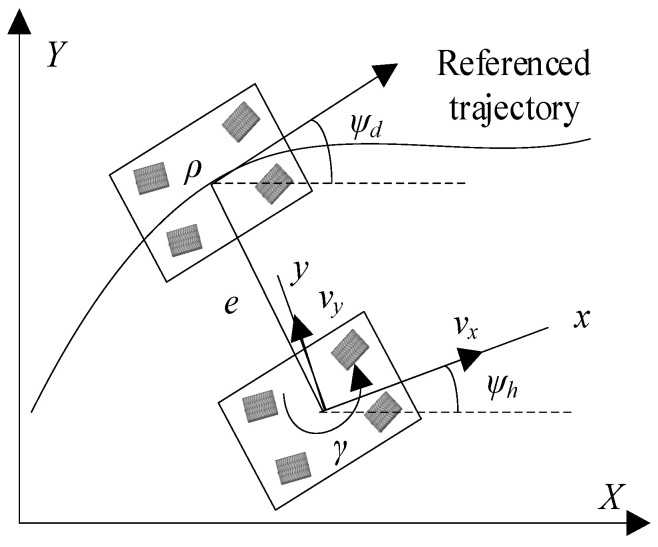
Schematic diagram of error model for unmanned vehicle trajectory tracking control.

Based on the principle of small-angle approximation, when the vehicle’s heading angle error is relatively small, the nonlinear kinematic relationship can be expanded by Taylor while retaining the first-order term, thereby establishing a simplified tracking error dynamic model. This model converts the three-dimensional spatial path error into a system of equations coupling the lateral deviation and the heading angle error in the two-dimensional plane by introducing the geometric projection relationship. In the path tracking dynamics model shown in Figure 2, the global geodetic coordinate system *XY* is defined by the Cartesian rectangular coordinate system. The *X*-axis extends along the centerline of the road, and the *Y*-axis is perpendicular to the *X*-axis and points to the left side of the vehicle, providing a standardized error feedback quantity reference for the subsequent controller design. It should be particularly noted that this error model decouples the parameters of the geometric deviation between the actual movement trajectory of the vehicle and the expected trajectory through coordinate system transformation. Among them, the lateral deviation e reflects the degree of position deviation of the vehicle in the direction perpendicular to the expected path, while the headcourse Angle error *ψ* quantifies the difference in the Angle between the vehicle’s headcourse and the tangent direction of the expected path. This two-degree-of-freedom error modeling method not only simplifies the design complexity of the control law but also provides a reliable performance evaluation index system for subsequent vehicle motion control.

The heading deviation and its differential equation can be expressed as

(7) $$\left\{\begin{array}{l} \psi =\psi_{h}-\psi_{d}\\ \dot{\psi }={\dot{\psi }}_{h}-{\dot{\psi }}_{d} \end{array}\right.,$$

where *ψ* represents the heading deviation, *ψ_h_* is the actual heading angle of the vehicle, and *ψ_d_* is the expected heading angle of the vehicle. *ψ_h_* and *ψ_d_* can be expressed as ${\dot{\psi }}_{h}=\gamma$ and ${\dot{\psi }}_{d}=\frac{v_{x}}{\rho }$, respectively.

(8) $$\dot{\psi }=\gamma -\frac{v_{x}}{\rho }$$

where *ρ* represents the radius of curvature of the expected path at the preview point. Using the Serret–Frenet equation, the lateral deviation equation of path tracking can be expressed as

(9) $$\dot{e}=v_{x}\sin\psi +v_{y}cos\psi +\gamma D_{p}$$

where *e* represents the lateral deviation and *D_p_* represents the aiming distance. Considering that the heading deviation angle is relatively small, Equation (9) can be simplified as

(10) $$\dot{e}=v_{x}\psi +v_{y}+\gamma D_{p}$$

By integrating the vehicle dynamics model and the trajectory tracking error model, the comprehensive model of unmanned vehicles that takes into account both stability control and trajectory tracking control can be obtained as follows

(11) $$\dot{x}=Ax+Bu+Ed$$

where the system state quantity can be expressed as $x={\left[\begin{array}{cccc} \psi & e & v_{y} & \gamma \end{array}\right]}^{T}$, the control input quantity is $u=\delta_{f}$, the uncertain input perturbation can be expressed as $d=\frac{1}{\rho }$, and the remaining system model parameter matrices are, respectively, $A=\left[\begin{array}{cccc} 0 & 0 & 0 & 1\\ v_{x} & 0 & 1 & D_{p}\\ 0 & 0 & \frac{C_{f}+C_{r}}{mv_{x}} & \frac{l_{f}C_{f}-l_{r}C_{r}}{mv_{x}}-v_{x}\\ 0 & 0 & \frac{l_{f}C_{f}-l_{r}C_{r}}{I_{z}v_{x}} & \frac{l_{f}^{2}C_{f}+l_{r}^{2}C_{r}}{I_{z}v_{x}} \end{array}\right]$, $B=\left[\begin{array}{c} 0\\ 0\\ -C_{f}/m\\ -l_{f}C_{f}/I_{z} \end{array}\right]$, $E=\left[\begin{array}{c} -v_{x}\\ 0\\ 0\\ 0 \end{array}\right]$.

### 2.3. Vehicle Multicellular Uncertainty Model Considering Parameter Perturbation

The uncertainty problem in the system modeling process is manifested in the objective differences between the theoretical mathematical model and the actual physical system. This inherent deviation will generate sustained disturbance effects through the input channel of the control system, leading to typical parameter uncertainty phenomena. Taking the vehicle autonomous driving tracking control system as an example, due to the nonlinear mechanical properties of tire rubber materials and the time-varying characteristics of longitudinal velocity, establishing an accurate vehicle dynamics model inherently involves unavoidable modeling errors. To solve this problem, it is usually necessary to perform multidimensional correction and compensation on the vehicle kinematic model in engineering practice.

When the vehicle is in a critical stable state, the contact between the tire tread and the ground will enter a nonlinear working zone. At this time, the traditional linear two-degree-of-freedom vehicle dynamics model can no longer effectively characterize the true motion response characteristics of the vehicle. Especially when the vehicle experiences a tendency to slide or performs emergency lane changes, the coupling effect of lateral acceleration and tire sideslip angle can lead to significant errors in the calculation of sideslip force. It is worth noting that the output characteristics of tire lateral force are not only affected by dynamic loads such as tire pressure fluctuations and changes in unsprung mass distribution, but also by the superposition of multiple uncertain factors such as sudden changes in road friction coefficient and tire surface wear, which results in strong random fluctuations in lateral force. In the controller algorithm design phase, a common approach to improve system robustness is to introduce a dynamic correction mechanism for tire lateral stiffness. Therefore, the improved mathematical expression for tire lateral stiffness can be written as

(12) $$\left\{\begin{array}{l} {\hat{C}}_{f}=C_{f}+k_{f}{\bar{C}}_{f}\\ {\hat{C}}_{r}=C_{r}+k_{r}{\bar{C}}_{r} \end{array}\right.,$$

where ${\hat{C}}_{f}$ and ${\hat{C}}_{r}$ are the correction values for the front and rear wheel lateral stiffness, ${\bar{C}}_{f}$ and ${\bar{C}}_{r}$ are the adjustment reference values for the front and rear wheel lateral stiffness, and *k_f_* and *k_r_* are the adjustment factors for the tire lateral stiffness, satisfying |*k_f_*|≤ 1 and |*k_r_*|≤ 1. To reduce computational complexity, *k_f_* = *k_r_* is set, which indicates the nonlinear characteristics of the tire.

The determination of the tire roll stiffness correction coefficient mainly depends on the variation law of parameters such as the tire roll angle and the road friction coefficient. This relationship can be obtained by establishing a mathematical model of the tire roll force and roll angle. When the friction coefficient of the road surface is taken as 0.85, it is usually assumed that the driving environment of the front and rear wheels of the vehicle is similar. At this time, the nonlinear characteristics of the lateral force-side deflection angle of the front and rear wheels can be regarded as the same. To reduce the computational complexity, based on the proportional relationship *k_f_* = *k_r_* set in Formula (12), the roll characteristics of the front and rear wheels can be uniformly characterized using the same curve model. By analyzing the corresponding relationship between the tire roll force and the roll angle, the value range of this correction coefficient is generally limited within the range of 0.2 to 1.0. The specific value can be dynamically adjusted in combination with the actual working conditions or the actual control effect of the vehicle. By substituting Equation (12) into Equation (11), we obtain

(13) $$\left\{\begin{array}{l} A(t)=A_{0}(t)+\Delta A(t)\\ B(t)=B_{0}(t)+\Delta B(t) \end{array}\right.,$$

where the model reference parameter matrices *A_0_* and *B_0_* constitute the nominal elements of the system coefficient matrix. The basic model, composed of these two types of parameters, is called the nominal system architecture, which is a theoretical model after eliminating potential uncertain factors through idealized assumptions. The parameter disturbance terms Δ*A* and Δ*B* are used to characterize various uncertain influences existing in the actual system, which are specifically reflected in the actual variation range of the tire side deflection stiffness parameter. This modeling method, by separating the nominal parameters from the disturbance quantities, not only retains the core dynamic characteristics of the system but also provides an effective mathematical framework for subsequent uncertainty analysis.

For vehicles with the common front-wheel steering configuration, since the rear wheels do not have a steering angle, the nonlinear characteristics of their tires are mainly manifested on the front wheels. In addition, when smart cars perform path tracking, the longitudinal speed changes dynamically. Considering that the longitudinal vehicle speed is generally bounded, it satisfies *V_x,min_* ≤ *V_x_* ≤ *V_x,max_*. Meanwhile, considering that the variation characteristics of the lateral stiffness of the front and rear tires are consistent, the time-varying variable of the parameter matrix at the vertex can be obtained as

(14) $$\left\{\begin{array}{l} C_{fr,\max}={\bar{\xi }}_{1},\;C_{fr,\min}={\bar{\xi }}_{2}\\ v_{x,\max}={\overset{⌢}{\xi }}_{1},\;v_{x,\min}={\overset{⌢}{\xi }}_{2}\\ 1/v_{x,\min}={\overset{⌣}{\xi }}_{1},\;1/v_{x,\max}={\overset{⌣}{\xi }}_{2} \end{array}\right.,$$

The selection strategy of the number of vertices in the multicellular structure has a direct impact on the computational complexity of the controller algorithm. Based on the exponential growth characteristic of the parameter space dimension, this study selects a hypercube structure with 2^3^ = 8 vertices and achieves complete coverage of the joint uncertainty domain of the vehicle dynamic parameter *v_x_*, its reciprocal term 1/*v_x_*, and the lateral stiffness coefficient *C_fr_* through geometric symmetry design. This multicellular modeling method adopts the weighted summation mechanism of vertex parameters for real-time estimation. Among them, the dynamic values of time-varying parameters *v_x_*, 1/*v_x,_* and *C_fr_* can be obtained through the linear combination operation of parameters at each vertex, which is specifically manifested as

(15) $$\left\{\begin{array}{l} C_{fr}=\sum_{j=1}^{3}{\bar{\sigma }}_{j}(t){\bar{\xi }}_{j}\\ v_{x}=\sum_{j=1}^{3}{\overset{⌢}{\sigma }}_{j}(t){\overset{⌢}{\xi }}_{j}\\ 1/v_{x}=\sum_{j=1}^{3}{\overset{⌣}{\sigma }}_{j}(t){\overset{⌣}{\xi }}_{j} \end{array}\right.$$

where $\sigma_{j}$ is the normalized weight coefficient and satisfies $\sum^{\sigma_{j}}$ = 1, *v*_*x*,*i*_, 1/*v*_*x*,*i*_, and *C*_*fr*,*I*_, respectively, represent the parameter values corresponding to the *i*-th vertex. This parametric representation method not only retains the essential characteristic of uncertainty of the original system, but also achieves an effective balance between computational complexity and accuracy through the finite-dimensional multi-cell structure. The normalized weight coefficient can be expressed in the following form

$$\left\{\begin{array}{l} {\bar{\sigma }}_{1}(t)=\frac{C_{fr,\max}-C_{fr}}{C_{fr,\max}-C_{fr,\min}},\;{\bar{\sigma }}_{2}(t)=\frac{C_{fr}-C_{fr,\min}}{C_{fr,\max}-C_{fr,\min}}\\ {\overset{⌢}{\sigma }}_{1}(t)=\frac{v_{x,\max}-v_{x}}{v_{x,\max}-v_{x,\min}},\;{\overset{⌢}{\sigma }}_{2}(t)=\frac{v_{x}-v_{x,\min}}{v_{x,\max}-v_{x,\min}}\\ {\overset{⌣}{\sigma }}_{1}(t)=\frac{1/v_{x,\min}-1/v_{x}}{1/v_{x,\min}-1/v_{x,\max}},\;{\overset{⌣}{\sigma }}_{2}(t)=\frac{1/v_{x}-1/v_{x,\max}}{1/v_{x,\min}-1/v_{x,\max}} \end{array}\right.$$

The normalized weight coefficients of the multicellular body can be combined to obtain

(16) $$\left\{\begin{array}{l} \sigma_{i}={\sigma^{\prime }}_{mnj}={\bar{\sigma }}_{m}{\overset{⌢}{\sigma }}_{n}{\overset{⌣}{\sigma }}_{j}\;\left(m=1,2\;n=1,2\;j=1,2\right)\\ \sigma_{1}={\sigma^{\prime }}_{111},\;\sigma_{2}={\sigma^{\prime }}_{112},\;\sigma_{3}={\sigma^{\prime }}_{121},\;\sigma_{4}={\sigma^{\prime }}_{122},\\ \sigma_{5}={\sigma^{\prime }}_{211},\;\sigma_{6}={\sigma^{\prime }}_{212},\;\sigma_{7}={\sigma^{\prime }}_{221},\;\sigma_{8}={\sigma^{\prime }}_{222}\\ \sum_{j=1}^{8}\sigma_{j}(t)=1,\;\sigma_{j}(t)⩾0 \end{array}\right.$$

Based on the above analysis, by integrating Equations (11)–(16), the trajectory tracking and dynamic control model of the intelligent vehicle convex multicellular body, considering the model uncertainty, can be expressed as

(17) $$\left\{\begin{array}{l} \dot{x}(t)=\sum_{j=1}^{8}\sigma_{j}(t)\left[A_{j}x\left(t\right)+B_{j}u\left(t\right)+E_{j}d\left(t\right)\right]\\ A_{j}=A_{0j}+\Delta A_{j},\;B_{j}=B_{0j}+\Delta B_{j} \end{array}\right.$$

According to the selected sampling time with a fixed discretization step size, Equation (17) is discretized. The discretized control model can ultimately be expressed as

(18) $$\left\{\begin{array}{l} x(k+1)=Ax(k)+Bu(k)+Ed(k)\\ y(k)=Cx(k)\\ A=\sum_{j=1}^{8}\sigma_{j}A_{j},B=\sum_{j=1}^{8}\sigma_{j}B_{j},E=\sum_{j=1}^{8}\sigma_{j}E_{j}\\ A,B,E\in \Omega \\ \Omega =C_{O}\left\{\left[A_{1}\left|B_{1}\right|E_{1}],\ldots ,[A_{8}\left|B_{8}\right|E_{8}\right]\right\} \end{array}\right.$$

where Ω is defined as the uncertain set of convex polycellular bodies, *Co* is the convex hull, and the state matrix and input matrix [*A_j_*, *B_j_*] are the vertices of the convex hull. This multicellular uncertain set is wrapped in a hypercube geometric form composed of vertex elements by wrapping the time-varying parameters in the system state space. As an important mathematical tool for describing the dynamic characteristics of linear variable parameter systems, the multi-cell model adopts a continuous variation characterization method of the parameter matrix in the time-varying constraint domain Ω(t)⊂*R*ⁿ×*P*, where *n* is the system state dimension and *P* is the parameter space dimension. The prediction model in Equation (17) achieves complete coverage of all possible evolution paths of the coefficient matrix in the infinite time domain by constructing a vertex update mechanism based on the instantaneous speed of the vehicle. This dynamic vertex generation strategy not only captures the time-varying characteristics of the parameter perturbation range but also establishes an error compensation mechanism by real-time reconstructing the vertex elements of the polycellular body to improve the characterization accuracy of the prediction model for the future system response, thereby effectively alleviating the impact of model mismatch on the robust control performance.

The optimization framework based on linear matrix inequalities (LMI) constructs an optimization problem with a strict mathematical basis by transforming the time-varying parameter constraints into the geometric description of convex sets. Specifically, this framework adopts the following transformation strategy: Firstly, the nonlinear inequality constraints of the original system are transformed into a linear form through variable substitution. Then, the coupling matrix is subjected to block triangulation processing by using Schur’s complement lemma. Finally, the entire optimization problem is standardized as a standard convex optimization problem. This transformation process not only maintains the integrity of the problem structure but also significantly improves the computational efficiency, making a real-time solution based on efficient numerical algorithms, such as the interior point method, possible, thereby providing a theoretical guarantee for the online optimization of robust controller parameters. It should be noted that the linear two-degree-of-freedom model proposed in this article and its combination with multi-cell uncertainty modeling have applicable boundaries, which are mainly suitable for small/medium side slip angles and medium/high adhesion coefficient conditions. For extreme working conditions such as large lateral deviation angles and low adhesion, its applicability may be reduced, requiring more complex nonlinear model support.

## 3. Robust Control Method for Trajectory Tracking with Parameter Uncertainty

### 3.1. Predictive Control of Min-Max Robust Model Based on LMI

The trajectory prediction architecture based on the kinematic relationship of vehicles integrates kinematic equations and linearized tire models. This model is mainly applicable to the dynamic description of vehicle motion under low-speed conditions or the analysis of scenarios that present linear relationships under specific parameter conditions. During the model construction process, if the perturbation effects of key parameters, such as the nonlinear time-varying characteristics of tire roll stiffness, are not fully considered, modeling deviations may lead to the risk of reduced trajectory prediction accuracy or even loss of vehicle stability. For such problems, the introduction of a vehicle dynamics model based on multi-cell parameter uncertainty analysis can more accurately capture the influence mechanism of parameter disturbances on the essential characteristics of the system under actual working conditions and establish an accurate parameter mapping relationship.

On this basis, a joint framework for vehicle trajectory tracking and dynamic control with multi-cell parameter uncertainties is constructed and combined with the design of a strong robust motion controller. This controller adopts the parameter region description method of convex multicellular bodies, geometrically characterizing the influence space of model parameter deviations and external disturbances, effectively reducing the negative impact of parameter mismatch on control performance. Specifically, based on the optimization design of Lyapunov stability theory and in combination with the parametric characteristics of the vertices of multicellular units, an adaptive control strategy capable of adapting to real-time parameter changes and unknown disturbances was constructed. This control architecture not only significantly improves the accuracy index of trajectory tracking but also enhances the driving stability and handling safety of the vehicle under complex working conditions by constructing a compact bounding domain with parameter variations.

For the discrete polycellular uncertain vehicle system equation in Equation (18), combined with the min-max optimization control idea, by adopting the optimization solution method of LMI, a robust controller for the uncertain vehicle system is constructed and the optimal robust control law for vehicle motion is obtained. On this basis, the predictive control problem of robust models for uncertain vehicle systems in the infinite time domain can be transformed into the problem of minimizing the objective function and solving the maximum tolerance boundary within a multi-cell uncertain set $\varphi_{z}$. The robust optimization control law of the final output, considering the perturbation of vehicle parameters and the uncertainty of the model, needs to ensure that the vehicle, under the premise of meeting the parameter output constraints, can suppress the trajectory tracking error value while taking into account the dynamic tracking control of the vehicle’s yaw velocity and lateral vehicle speed for the reference value. Thus, the min-max optimization solution problem that takes into account both the minimization of the objective function and the setting of the maximum tolerance boundary can be expressed as

(19) $$\left\{\begin{array}{l} A(\rho )=A_{0}+\Delta A_{0}\\ B(\rho )=B_{0}+\Delta B_{0} \end{array}\right.,$$

where *Q_x_* represents the state weight matrix and *R_u_* represents the control weight matrix. Analysis shows that the infinite time domain optimization problem covered in Equation (19) is usually difficult to solve directly. At this time, it is necessary to obtain the upper bound that can represent the performance index of the objective function, so that the optimization problem can be equivalent to a minimization optimization solution problem under the condition of a finite number of decision variables. Therefore, in order to effectively represent the upper bound of the objective function, the quadratic Lyapunov–Krasovskii function is selected and defined as follows

(20) $$V(x)=\delta x^{T}Px,\;\;P>0,\;\;P=P^{T},$$

Meanwhile, V(x) must satisfy the robust constraints characterized by the following inequality for any $\begin{array}{c} \left[\begin{array}{c} A_{k+t},B_{k+t} \end{array}\right] \end{array}\in \varphi_{z},t\geq 0$ within the sampling time *k*

(21) $$\begin{array}{l} V[x(k+t+1|k)]-V[x(k+t|k)]⩽\\ -\left[x^{T}(k+t|k)Q_{x}x(k+t|k)+u^{T}(k+t|k)R_{u}u(k+t|k)\right] \end{array},$$

To ensure that the performance function of the objective function is bounded, it is necessary to satisfy the condition that the control system state tends infinitely to zero at an infinite number of moments, and the system can still meet the closed-loop asymptotic stability condition. Its expression is

(22) $$V[x(\infty |k)]=x_{\infty }^{T}Px_{\infty }=0,$$

Equation (21) can be obtained by accumulating from *t* = 0 to *t* = ∞

(23) $$\begin{array}{l} V[x(\infty |k)]+\ldots +V[x(k+2|k)]-V[(k+1|k)]+V[x(k+1|k)]-V[x(k|k)]\\ ⩽-[x^{T}(k|k)Q_{x}x(k|k)+\;u^{T}(k|k)R_{u}u(k|k)]-[x^{T}(k+1|k)\times Q_{x}x(k+1|k)+\;\\ u^{T}(k+1|k)\times R_{u}u(k+1|k)]-\ldots -x^{T}(\infty |k)\times \;Q_{x}x(\infty |k)+u^{T}(\infty |k)R_{u}u(\infty |k)\; \end{array},$$

Combining Equation (22), it can be known that at infinite moments, the first term on the left side of the inequality in Equation (23) approaches 0; thus, Equation (23) can be expressed as

(24) $$-V[x\left(k\left|k\right.\right)]⩽-J_{\infty }(k),$$

The minimization mechanism of performance metrics is equivalent to the minimization of $V[x\left(k\left|k\right.\right)]$. At this point, the upper bound of the maximum tolerance boundary can be expressed as

(25) $$\max J_{\infty }(k)⩽V[x(k|k)]=\tau ,$$

Under the action of the robust controller for vehicle trajectory tracking, within a single sampling period, the robust feedback control law based on the optimization solution of LMI inequalities minimizes the upper limit value of the quadratic Lyapunov–Krasovskii function. In the subsequent sampling period iteration process, by combining the dynamic feedback update results of the state, the optimization solution and iterative update of the objective function are achieved, thereby obtaining the global feedback control law. If we select $P=\tau \theta^{-1}$ and $\psi =H\theta^{-1}$, the state feedback control law of the vehicle trajectory tracking robust controller can be expressed as

(26) $$u\left(\left.k+t\right|k\right)=\psi x\left(\left.k+t\right|k\right),$$

The LMI inequality corresponding to the min-max problem in Equation (19) can be expressed as

(27) $$\left[\begin{array}{cc} 1 & *\\ x\left(k\right) & \theta \end{array}\right]⩾0,$$

where * represents the transpose matrix of the diagonal elements in the same matrix. Among them, the above formula can restrict the vehicle state within a single sampling period to the elliptic invariant set *θ*, and its constraint conditions can be expressed as

(28) $$\left[\begin{array}{cccc} \theta & * & * & *\\ A_{zl}\theta +B_{zl}H & \theta & 0 & 0\\ \sqrt{Q_{x}}\theta & 0 & \tau I & 0\\ \sqrt{R_{u}}H & 0 & 0 & \tau I \end{array}\right]⩾0,$$

The above equation, while conforming to the constraint convergence of the Lyapunov–Krasovskii function, can effectively match the upper bound τ of the maximum tolerance boundary of the optimization function. The input constraints and output constraints in the optimization solution process can be expressed as

(29) $$\left[\begin{array}{cc} u_{max^{2}}I & *\\ H^{T} & \theta \end{array}\right]⩾0,$$

(30) $$\left[\begin{array}{cc} \theta & *\\ C(A_{zl}\theta +B_{zl}H) & y_{max^{2}}I \end{array}\right]⩾0,\;l\;=\;1\;,2\;,\ldots ,8,$$

Therefore, the optimization solution problem of the robust controller for vehicle trajectory tracking based on LMI can be comprehensively expressed as

(31) $$\begin{array}{c} \underset{\tau ,\theta ,H}{\min}\tau \\ s.t.\;\left(27\right),\left(28\right),\left(29\right),\left(30\right) \end{array},$$

### 3.2. Predictive Control of Min-Max Robust Model Based on Improved LMI

In a robust model predictive controller, the control invariant set provides a mechanism to ensure that the system state remains within a specific set when affected by disturbances and uncertainties, thereby guaranteeing the stability of the control system. By designing a reasonable control invariant set, the conservatism of the control strategy can be reduced and the feasible region of the system can be expanded, thereby enhancing the control effect and making the system response more rapid.

**Figure 3:**
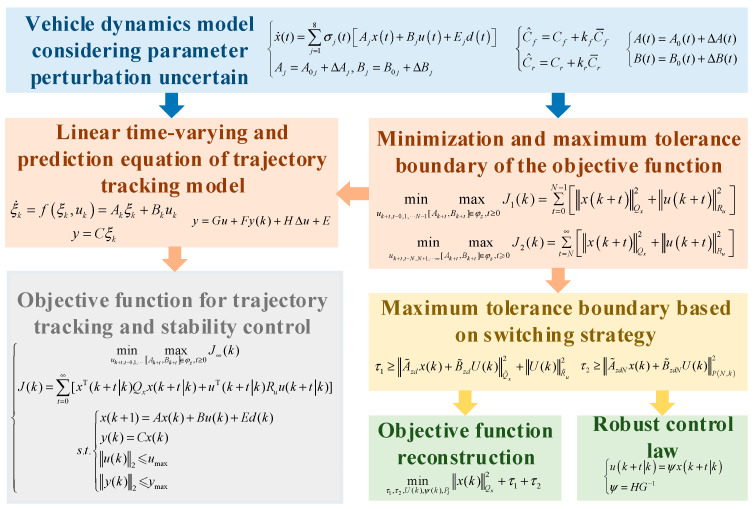
Overall architecture diagram of min-max RMPC system based on improved LMI.

In robust model predictive control, by introducing a control invariant set, the allowable area of the initial state can be expanded, that is, more initial states can be controlled onto the desired trajectory. The control invariant set can be used to handle the disturbances that the system is subjected to. By designing a robust controller, the system can maintain its performance when facing disturbances, and even guide the system state to the minimum robust positive invariant set. In addition, by rationally designing the control invariant set, part of the online computational load can be transferred to the offline design for completion, effectively reducing the online computational load and improving the efficiency of the robust predictive controller. The overall structure of the min-max robust model predictive control system based on the improved LMI is shown in Figure 3.

In the optimization solution process of the objective function in Equation (31), the system state is restricted within the control invariant set *θ* in the overall control process. Therefore, it is difficult to optimize the vehicle control effect by contracting the terminal boundary of the control set. It is hard for this setting method to fully meet the strict requirements for high dynamic and high reliability performance of intelligent vehicles during actual driving. During the actual driving process of intelligent vehicles, if the terminal boundary conditions of the control invariant set are selected too widely, it will directly lead to the vehicle trajectory tracking robust controller being insufficiently sensitive to the dynamic changes of parameter perturbation and model uncertainty. At this point, the robust controller is overly conservative in setting the model’s uncertainties, thereby losing, to some extent, the dynamic robustness of the vehicle controller when implementing trajectory tracking and stability control. Furthermore, if the terminal boundary conditions of the control invariant set are selected too strictly, the number of vertices in the polycellular set will increase sharply, thereby leading to a sharp increase in the demand for optimization solution operations of the robust controller. In this case, for high-order nonlinear vehicle systems, the sharply increased computational load will, to some extent, affect the accuracy and real-time performance of vehicle dynamic control. Especially when it comes to complex intelligent driving scenarios and dynamic changing working conditions with high vehicle speeds, the parameter perturbation and uncertainty of the vehicle system model also have high dynamic change characteristics. At this time, the vehicle robust controller has stricter limitations on the computational load for optimization solutions. Therefore, a reasonable robust controller is crucial for enhancing the overall control performance of a vehicle.

To solve this problem and further improve the vehicle control performance, the optimization design of the robust controller was carried out in two steps based on the switching strategy. We construct N control law variables corresponding to the system control output and incorporate them into the vehicle’s robust feedback control law, that is

(32) $$u\left(\left.k+t\right|k\right)=\left\{\begin{array}{ll} u\left(\left.k\right|k\right), & t=0\\ u\left(\left.k+1\right|k\right), & t=1\\ \vdots \\ \varphi x\left(\left.k+t\right|k\right), & t⩾N \end{array}\right.,$$

Based on Equation (19), the solution methods for minimizing the objective function and the maximum tolerance boundary are reconstructed and decomposed into the following two sub-terms

(33) $$\underset{u_{k+t,t-0,\;1,\;\ldots N-1}[A_{k+t},B_{k+t}]\in \varphi_{z},t\geq 0}{\;\;\;\min\;\;\;\;\;\;\;\max}J_{1}(k)=\sum_{t=0}^{N-1}\left[{\left\|x\left(k+t\right)\right\|}_{Q_{x}}^{2}\begin{array}{c} +{\left\|u\left(k+t\right)\right\|}_{R_{u}}^{2} \end{array}\right],$$

(34) $$\underset{u_{k+t,t-N,N+1,\ldots \infty }[A_{k+t},B_{k+t}]\in \varphi_{z},t⩾0}{\;\;\;\min\;\;\;\;\;\;\;\max}J_{2}(k)=\sum_{t=N}^{\infty }\left[\begin{array}{c} {\left\|x\left(k+t\right)\right\|}_{Q_{x}}^{2}+{\left\|u\left(k+t\right)\right\|}_{R_{u}}^{2} \end{array}\right],$$

Meanwhile, the optimization objective functions in Equations (33) and (34) also need to satisfy all the constraints in Equation (19). Therefore, based on the reconstructed objective function, the time-varying Lyapunov function for can be modified to

(35) $$V(t,k)=x{\left(k+t\right)}^{T}P(t,k)x(k+t),\;t⩾N,$$

At this point, the reconstructed Lyapunov function also needs to satisfy the following robust inequality constraints for any $\left[\begin{array}{c} A_{k+t},B_{k+t} \end{array}\right]\in \varphi_{z}\left(t⩾0\right)$

(36) $$V(t+1,k)-V(t,k)⩽-{\left\|x\left(\left.k+t\right|k\right)\right\|}_{Q_{s^{x}}}^{2}-{\left\|u\left(\left.k+t\right|k\right)\right\|}_{R_{u}}^{2},$$

Therefore, in the same way, Equation (36) can be obtained by accumulating from *t* = *N* to *t* = ∞

(37) $$\begin{array}{l} V(\infty ,k)+V(N+2,k)-V(N+1,k)+V(N+1,k)-V(N,k)\\ ⩽-{\left\|x\left(\left.k+N\right|k\right)\right\|}_{Q_{s^{x}}}^{2}-{\left\|u\left(\left.k+N\right|k\right)\right\|}_{R_{u}}^{2}-{\left\|x\left(\left.k+N+1\right|k\right)\right\|}_{Q_{s^{x}}}^{2}\\ -{\left\|u\left(\left.k+N+1\right|k\right)\right\|}_{R_{u}}^{2}-{\left\|x\left(\left.k+N+\infty \right|k\right)\right\|}_{Q_{s^{x}}}^{2}-{\left\|u\left(\left.k+N+\infty \right|k\right)\right\|}_{R_{u}}^{2} \end{array},$$

It can be observed that all the sub-terms on the right side of inequality (37) are equivalent to $-J_{2}(k)$, and thus, we can obtain

(38) $$-V(N,k)⩽-J_{2}(k),$$

Therefore, the infinite time domain optimal problem corresponding to Equation (38) can be transformed into the problem of minimizing the upper bound of the maximum tolerance boundary, that is

(39) $$\max J_{2}(k)⩽V(N,k)=x{\left(k+N\right)}^{T}P(t,k)x(k+N),$$

Meanwhile, the objective function in Equation (33) is equivalent to achieving the transformation of the optimization objective function from the infinite time domain to the finite time domain, that is

(40) $$\max{\tilde{J}}_{\infty }(k)⩽\sum_{t=0}^{N-1}\left[{\left\|x\left(k+t\right)\right\|}_{Q_{x}}^{2}+{\left\|u\left(k+t\right)\right\|}_{R_{u}}^{2}\right]+V(N,k),$$

In Equation (40), the right sub-term is equivalent to the terminal boundary condition in the model’s predictive controller. Therefore, the performance index function of the extended robust model predictive controller can be designed in the following form

(41) $$\tilde{J}(k)=\sum_{t=0}^{N-1}\left[{\left\|x\left(\left.k+t\right|k\right)\right\|}_{Q_{s^{x}}}^{2}+{\left\|u\left(\left.k+t\right|k\right)\right\|}_{R_{u}}^{2}\right]+{\left\|x\left(\left.k+N\right|k\right)\right\|}_{P\left(N,k\right)}^{2},$$

Combining the vehicle convex multicellular body trajectory tracking and dynamic control model in Equation (18), the state prediction matrix within an *N*-step sampling period is

(42) $$\left[\begin{array}{c} x(k+1)\\ x(k+2)\\ \vdots \\ x(k+N) \end{array}\right]=\left[\begin{array}{c} A_{zd}\\ A_{zd}^{2}\\ \vdots \\ A_{zd}^{N} \end{array}\right]x(k)+\left[\begin{array}{cccc} B_{zd} & 0 & \ldots & 0\\ A_{zd}B_{zd} & B_{zd} & \ldots & 0\\ \vdots & \vdots & & 0\\ A_{zd}^{N-1}B_{zd} & \ldots & \ldots & B_{zd} \end{array}\right]\times \left[\begin{array}{c} u(k)\\ u(k+1)\\ \vdots \\ u(k+N-1) \end{array}\right],$$

Equation (42) can be simplified to the following block matrix expression form

(43) $$\left[\begin{array}{c} \tilde{x}(k+1)\\ x(k+N) \end{array}\right]=\left[\begin{array}{c} {\tilde{A}}_{zd}\\ {\tilde{A}}_{zdN} \end{array}\right]x(k)+\left[\begin{array}{c} {\tilde{B}}_{zd}\\ {\tilde{B}}_{zdN} \end{array}\right]U(k),$$

where $\tilde{x}(k+1)$, U(k), ${\tilde{A}}_{zd}$, ${\tilde{B}}_{zd}$, ${\tilde{A}}_{zdN}$, ${\tilde{B}}_{zdN}$ corresponds to the set of block matrices in Equation (42). Therefore, the performance index function of the robust controller can be transformed into

(44) $$\tilde{J}(k)={\left\|x\left(k\right)\right\|}_{Q_{x}}^{2}+{\left\|{\tilde{A}}_{zd}x\left(k\right)+{\tilde{B}}_{zd}U(k)\right\|}_{{\tilde{Q}}_{x}}^{2}+{\left\|U\left(k\right)\right\|}_{{\tilde{R}}_{u}}^{2}+{\left\|{\tilde{A}}_{zdN}x\left(k\right)+{\tilde{B}}_{zdN}U(k)\right\|}_{P\left(N,k\right)}^{2},$$

where ${\tilde{Q}}_{x}=diag\left[Q_{x},Q_{x},\ldots ,Q_{x}\right]$, ${\tilde{R}}_{u}=diag\left[R_{u},R_{u},\ldots ,R_{u}\right]$, both ${\tilde{R}}_{u}$ and ${\tilde{R}}_{u}$ are block diagonal arrays, and the augmented dimension is directly related to the size of the number of control variables *N*. Based on the above analysis, it can be known that if the vehicle robust feedback control law and robust inequality constraints in Equations (42) and (36) are to be satisfied, it is necessary to ensure that the unique *L* symmetric positive definite matrices *p*_1_ = (1,2,… *L*), such that the following conditions are fully satisfied

(45) $$\begin{array}{l} P\left(t,k\right)=\sum_{l=1}^{8}\beta_{l}\left(k+t\right)p_{l}{\left[A_{zl}+B_{zl}\psi \left(k\right)\right]}^{T}P_{t}\left[A_{zl}+B_{zl}\psi \left(k\right)\right]\\ -P_{l}+Q_{x}+\psi^{T}\left(k\right)R_{u}\psi \left(k\right)\leq 0,\;\;\;l,t=1,2,\ldots ,8 \end{array},$$

On this basis, the upper bound of the maximum tolerance boundary based on the switching strategy is redesigned as

(46) $$\tau_{1}\geq {\left\|{\tilde{A}}_{zd}x(k)+{\tilde{B}}_{zd}U(k)\right\|}_{{\tilde{Q}}_{x}}^{2}+{\left\|U(k)\right\|}_{{\tilde{R}}_{u}}^{2},$$

(47) $$\tau_{2}\geq {\left\|{\tilde{A}}_{zdN}x(k)+{\tilde{B}}_{zdN}U(k)\right\|}_{P\left(N,k\right)}^{2},$$

Therefore, the objective function that takes into account both the trajectory tracking and stability optimization control problems of intelligent convex multi-cell vehicles can be designed as

(48) $$\underset{\tau_{1},\tau_{2},U(k),\psi (k),P_{l}}{\min}{\left\|x(k)\right\|}_{Q_{x}}^{2}+\tau_{1}+\tau_{2},$$

Based on Shul’s complement theorem, the optimization objective function constraint problems from Equation (45) to Equation (47) can be transformed into the LMI form. By defining $\theta_{l}=\tau_{2}P_{l}^{-1}$ and $\left[\begin{array}{cccc} G+G^{T}-\theta_{l} & * & * & *\\ A_{zl}G+B_{zl}H & \theta_{j} & * & *\\ Q_{x}G & 0 & \tau_{2}I & *\\ R_{u}H & 0 & 0 & \tau_{2}I \end{array}\right]⩾0,l,j=1,2,\ldots ,8$, then Equation (45) can be expressed as

(49) $$\left[\begin{array}{cccc} G+G^{T}-\theta_{l} & * & * & *\\ A_{zl}G+B_{zl}H & \theta_{j} & * & *\\ Q_{x}G & 0 & \tau_{2}I & *\\ R_{u}H & 0 & 0 & \tau_{2}I \end{array}\right]⩾0,l,j=1,2,\ldots ,8,$$

In the modeling process, for the multicellular system architecture, $\left[{\tilde{A}}_{zd}\;\;{\tilde{B}}_{zd}\right]$ can be extended to the following multicellular expression form in combination with Equation (42)

(50) $$\left\{\begin{array}{l} \left[\begin{array}{cc} {\tilde{A}}_{zd} & {\tilde{B}}_{zd} \end{array}\right]\in \varphi_{zd}\\ \varphi_{zd}=C_{0}\left\{\left[{\tilde{A}}_{z1}\;{\tilde{B}}_{z1}\right],\left[{\tilde{A}}_{z2}\;{\tilde{B}}_{z2}\right],\ldots ,\left[{\tilde{A}}_{z8}\;{\tilde{B}}_{z8}\right]\right\} \end{array}\right.,$$

(51) $$\left[\begin{array}{cc} {\tilde{A}}_{zd} & {\tilde{B}}_{zd} \end{array}\right]=\sum_{l=1}^{8}\kappa_{l}\left[\begin{array}{cc} {\tilde{A}}_{zl} & {\tilde{B}}_{zl} \end{array}\right],\;\sum_{l=1}^{8}\kappa_{l}=1,\;\kappa_{l}⩾0,$$

The upper bound of the maximum tolerance boundary $\tau_{1}$ of the robust controller can be transformed into the following LMI matrix form by using Equation (46)

(52) $$\left[\begin{array}{ccc} {\tilde{Q}}_{x}^{-1} & * & *\\ 0 & {\tilde{R}}_{u}^{-1} & *\\ x{\left(k\right)}^{T}{\tilde{A}}_{zl}+U{\left(k\right)}^{T}{\tilde{B}}_{zl} & U{\left(k\right)}^{T} & \tau_{1}I \end{array}\right]⩾0,l=1,2,\ldots ,8,$$

Similarly, the terminal augmentation matrices ${\tilde{A}}_{zdN}$ and ${\tilde{B}}_{zdN}$ in Equation (47) can be transformed into the following multicellular expression form

(53) $$\left\{\begin{array}{l} \left[\begin{array}{cc} {\tilde{A}}_{zdN} & {\tilde{B}}_{zdN} \end{array}\right]\in \varphi_{zdN}\\ \varphi_{zdN}=C_{o}\left\{\left[{\tilde{A}}_{z1N}\;{\tilde{B}}_{z1N}\right],\left[{\tilde{A}}_{z2N}\;{\tilde{B}}_{z2N}\right],\ldots ,\left[{\tilde{A}}_{z8N}\;{\tilde{B}}_{z8N}\right]\right\} \end{array}\right.,$$

(54) $$\left\{\begin{array}{l} \left[{\tilde{A}}_{zdN}\;{\tilde{B}}_{zdN}\right]=\sum_{l=1}^{8}\gamma_{l}\left[\begin{array}{cc} {\tilde{A}}_{z1N} & {\tilde{B}}_{z1N} \end{array}\right],\\ \sum_{l=1}^{8}\gamma_{l}=1,\;\gamma_{l}⩾0 \end{array}\right.,$$

On this basis, the upper bound of the maximum tolerance boundary $\tau_{2}$ of the robust controller in Equation (47) can be transformed into the following LMI matrix form

(55) $$\left[\begin{array}{cc} 1 & *\\ {\tilde{A}}_{zlN}x{\left(k\right)}^{T}+{\tilde{B}}_{zlN}U{\left(k\right)}^{T} & \theta_{l} \end{array}\right]⩾0,l=1,2,\ldots ,8,$$

At this point, the input and output constraints of the optimization objective function are

(56) $$\left[\begin{array}{cc} u_{\max}^{2}I & *\\ H^{T} & G+G^{T}-\theta_{l} \end{array}\right]⩾0,\;l=1,2,\ldots ,8,$$

(57) $$\left[\begin{array}{cc} G+G^{T}-\theta_{l} & *\\ C\left(A_{zd}G+B_{zd}H\right) & y_{\max}^{2}I \end{array}\right]⩾0,\;l=1,2,\ldots ,8,$$

Based on the above constraint condition formulas, the optimization solution problem of the improved vehicle robust model predictive controller can be equivalent to

(58) $$\begin{array}{c} \underset{\tau_{1},\tau_{2},U(k),H,G,\theta_{l}}{\min}{\left\|x(k)\right\|}_{Q_{x}}^{2}+\tau_{1}+\tau_{2}\\ s.t.\;\left(29\right),\left(32\right),\left(35\right),\left(36\right),\left(37\right) \end{array},$$

At this point, the robust output control law of the improved vehicle robust model predictive controller can be expressed as

(59) $$\left\{\begin{array}{l} u\left(k+t\left|k\right.\right)=\psi x\left(k+t\left|k\right.\right)\\ \psi =HG^{-1} \end{array}\right.,$$

## 4. Simulation Results

To verify the adaptability and robustness of the proposed robust model predictive control algorithm under parameter uncertainty conditions, this subsection first designs a set of contrastive simulation tests under high-adhesion and low-speed conditions. In the simulation, to characterize the uncertainty of the model parameters, two different test conditions with a 10% parameter perturbation deviation and a 30% parameter perturbation deviation were, respectively, set. By setting the deviation range of the vehicle dynamics model parameters, the robustness against parameter uncertainty under different parameter perturbations and the influence of the corresponding control algorithm on the vehicle dynamics control effect are simulated and reflected. In addition, to ensure the consistency of the test conditions, the road adhesion coefficient was set to 1.0 and the vehicle traveling speed to 10 m/s in the joint simulation.

**Figure 4:**
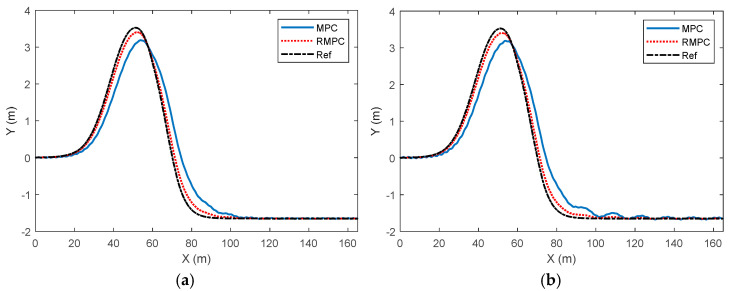
Comparison of vehicle trajectory tracking control effects under high-adhesion low-speed driving conditions. (**a**) Parameter perturbation deviation of 10%, (**b**) parameter perturbation deviation of 30%.

Considering the uncertainty of model parameters, the comparison results of vehicle trajectory tracking control effects under high-attachment and low-speed conditions are shown in Figure 4. It can be seen from the figure that although the trajectory under the MPC algorithm is roughly consistent with the change trend of the reference value, there is a certain deviation compared with the reference trajectory near the peak and during the decline stage, indicating that there are certain limitations in tracking accuracy and amplitude control. In contrast, the trend changes of the trajectory under the RMPC algorithm are closer to those of the reference trajectory, especially during the stage when the lateral displacement rises to the peak and then drops rapidly, that is, when the vehicle changes lanes quickly. The actual driving trajectory has a higher degree of overlap with the reference value, demonstrating higher tracking accuracy and real-time performance. When the degree of parameter perturbation deviation changes from 10% to 30%, the vehicle driving trajectory under the MPC algorithm is significantly affected by the model uncertainty. However, in this case, the RMPC does not show a significantly affected trend and can still maintain a good control effect. Overall, RMPC outperforms traditional MPC methods in terms of trajectory change trends, amplitude magnitudes, and real-time tracking performance of reference trajectories, demonstrating stronger dynamic response capabilities and higher control accuracy. This indicates that RMPC has better robustness when facing larger system parameter uncertainties.

**Figure 5:**
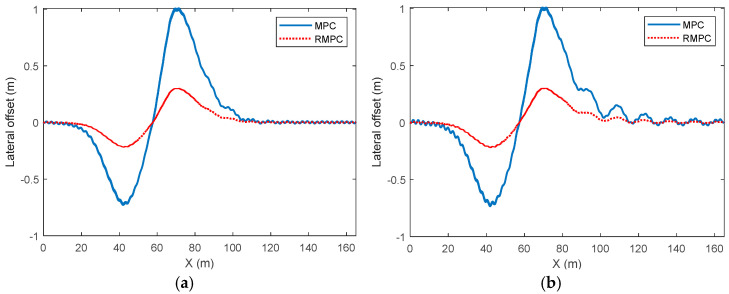
Comparison of lateral deviation in vehicle trajectory tracking control under high-adhesion low-speed driving conditions. (**a**) Parameter perturbation deviation of 10%, (**b**) parameter perturbation deviation of 30%.

The comparison results of the lateral deviation of vehicle trajectory tracking control under high-attachment and low-speed conditions are shown in Figure 5. As can be seen from the figure, there is a significant difference in the lateral deviation of trajectory tracking under MPC and RMPC. The maximum value of the lateral deviation under MPC is close to 1 m, while under the same conditions, the maximum value of the lateral deviation under RMPC is approximately 0.2 m. The significant increase in lateral deviation mainly occurs during the process of vehicle lane changing. During the vehicle’s return to the center stage, the lateral deviation gradually decreases and approaches zero. At this point, compared with MPC, the lateral deviation under the RMPC algorithm can tend to stabilize more quickly and converge to 0.

**Figure 6:**
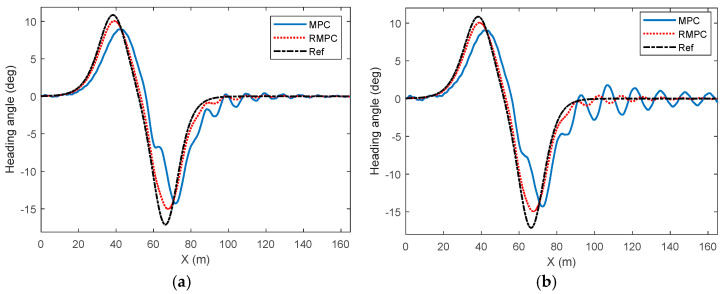
Comparison of heading angle in vehicle trajectory tracking control under high-adhesion low-speed driving conditions. (**a**) Parameter perturbation deviation of 10%, (**b**) parameter perturbation deviation of 30%.

**Figure 7:**
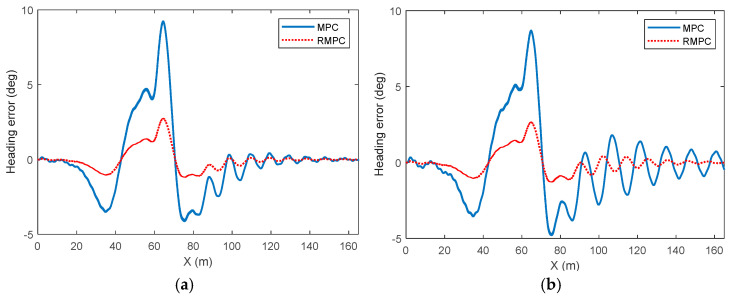
Comparison of heading deviation in vehicle trajectory tracking control under high-adhesion low-speed driving conditions. (**a**) Parameter perturbation deviation of 10%, (**b**) parameter perturbation deviation of 30%.

Considering the uncertainty of model parameters, the comparison results of the heading angle for vehicle trajectory tracking control under high-attachment low-speed conditions are shown in Figure 6. Analysis shows that when there is uncertainty in model parameters, the dynamic change trend of the heading angle under different degrees of parameter mismatch has a strong correlation with the deviation trend of the vehicle trajectory in Figure 4. In Figure 6a, the RMPC algorithm can effectively deal with the uncertainty of model parameters and achieve dynamic tracking of the reference heading angle as a whole. Especially when the heading angle undergoes drastic changes, the tracking real-time performance of the RMPC algorithm is significantly better than that of the MPC algorithm. When the vehicle trajectory returns to alignment and tends to stabilize, that is, when the reference heading angle is 0, the heading angle under the RMPC algorithm can converge rapidly and remain stable, while the heading angle under the MPC algorithm shows obvious fluctuations near the zero value, and the degree of fluctuation gradually increases with the increase of the parameter perturbation deviation degree. This indicates that the MPC algorithm is highly sensitive to the perturbation of model parameters. As the uncertainty of model parameters increases, its control performance gradually declines. However, at this time, the RMPC algorithm shows stronger robustness when dealing with parameter uncertainties, and its control performance does not show significant changes when the deviation degree of model parameter perturbation increases. The comparison results of heading deviations for vehicle trajectory tracking control under high-attachment and low-speed conditions are shown in Figure 7. Based on the content in the figure, it can be known that under the influence of model uncertainty, the maximum heading angle deviation under MPC reaches about 9 deg, while under RMPC, the heading angle deviation basically does not exceed 2 deg. This further reflects the role of RMPC in heading control.

**Figure 8:**
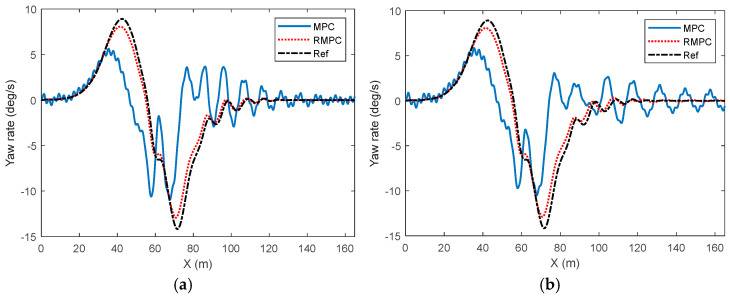
Comparison of yaw rate in vehicle trajectory tracking control under high-adhesion low-speed driving conditions. (**a**) Parameter perturbation deviation of 10%, (**b**) parameter perturbation deviation of 30%.

The comparison results of the yaw velocity of vehicle trajectory tracking control under high-attachment low-speed conditions are shown in Figure 8. The yaw velocity curve under the MPC algorithm shows a relatively obvious lag when tracking the reference value, especially near the peak of the reference value. The response of MPC is relatively slow and cannot quickly follow the demand changes of the reference yaw velocity. In contrast, the yaw control curve under the RMPC algorithm demonstrates a higher response speed and control accuracy during tracking control. The actual vehicle yaw is in perfect agreement with the reference value, demonstrating excellent dynamic performance. In addition, during the rapid lane-changing process of vehicles, the real-time status of the vehicles changes rapidly. The dynamic characteristics change more complexly after being affected by the uncertainty of model parameters. Therefore, at this time, the overall change trend of the actual vehicle yaw velocity curve under the MPC algorithm and the actual amplitude fluctuation size have deviated significantly from the reference value. With the increase of parameter perturbation degree, the fluctuation and oscillation degree of the actual vehicle yaw velocity during the trajectory correction process under the MPC algorithm gradually increase. However, the RMPC algorithm can maintain relatively good tracking performance and can maintain good control effects in terms of fluctuation amplitude and convergence speed.

## 5. Experimental Results

**Figure 9:**
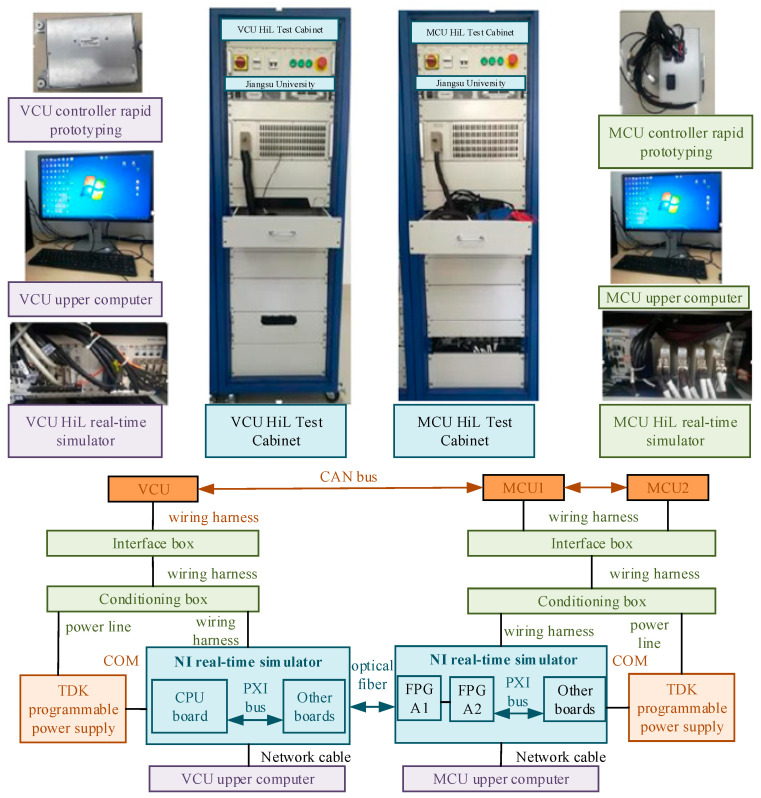
HiL test platform architecture.

Figure 9 shows the system architecture of the hardware-in-the-loop simulation platform. The core hardware components of this architecture include the upper computer for the vehicle control unit (VCU) and the motor control unit (MCU), the test cabinet, and the rapid prototyping controller, manufactured by NI Corporation in Austin, TX, USA. The VCU and MCU test cabinets adopt a rack-mounted design layout. The integration sequence is as follows: power management module, programmable power supply, distribution box, interface box, signal conditioning box, and NI real-time simulator (PXI 85132, PXI 7811R, PXI 6723). The vehicle dynamics model is deployed on the NI real-time simulator processor board on the VCU side. The output signal of this model is transmitted to the VCU via a hardwire interface. After receiving the signal, the VCU calculates the steering and driving instructions necessary for the motion control of the unmanned vehicle and sends these instructions to the actuator module. The HiL test was conducted using a NI real-time simulator with a control cycle of 10 ms. Therefore, all simulation and test results were run based on this cycle.

To verify the implementation effect of the developed control strategy in the actual controller, we integrated the existing software and hardware resources and constructed a modular hardware-in-the-loop test platform. This platform comprehensively covers the systematic development of vehicle dynamics models, hub motor models, motor controllers, and related control algorithms. Under the hardware-in-the-loop simulation architecture, vehicle dynamics models are used to simulate the comprehensive performance of vehicles in different test scenarios. This experiment adopts the VCU HiL test device. The management software VeriStand 2020 equipped in it interacts with the model through control signals and invokes the model. Therefore, the vehicle model needs to be compiled and processed into a recognizable dynamic link library (.dll) format. In the specific implementation process, a discretized vehicle dynamics model was constructed based on the Simulink environment, and a fixed-step discretization solution strategy was adopted. The output interface of the model is configured in a form compatible with the VeriStand communication protocol. Ultimately, the compiled.DLL file was deployed to the real-time simulator, thus achieving the goal of joint testing and providing strong support for the verification of the effectiveness of the control strategy.

To verify the adaptability and robustness of the proposed robust model predictive control algorithm under parameter uncertainty conditions, a comparative simulation test under high-adhesion and low-speed conditions was conducted in combination with the HiL test platform. In the simulation, a test condition with a 20% perturbation deviation of parameters was set, the road adhesion coefficient was 1.0, and the vehicle traveling speed was 10 m/s.

**Figure 10:**
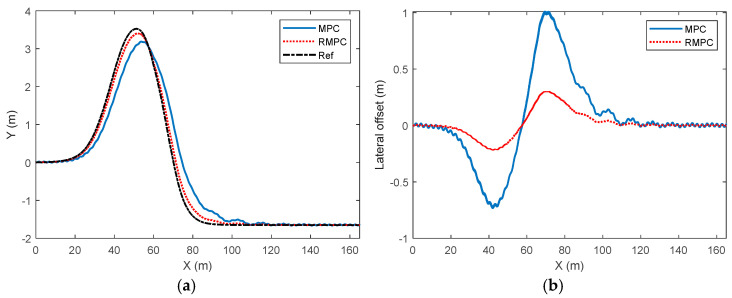
HiL test results for vehicle trajectory tracking control under high- and low-speed operating conditions. (**a**) Vehicle trajectory, (**b**) lateral deviation.

Figure 10 reflects the performance comparison results of vehicle trajectory tracking control under high-adhesion and low-speed conditions, under the condition of uncertainty of model parameters. The results show that although the driving path generated by the MPC strategy is basically in line with the expected path change trend, there are obvious deviations in both the peak and attenuation intervals, revealing its limitations in trajectory tracking accuracy and amplitude control. In comparison, the output trajectory of the RMPC strategy shows a higher consistency with the reference values. Especially in the rapid lane-changing condition where the lateral displacement rises sharply to the peak and then drops rapidly, the actual driving path is highly consistent with the reference trajectory, effectively verifying the superior performance of this strategy in improving tracking accuracy and real-time dynamic response.

According to the comparison results of the lateral deviation of vehicle trajectory tracking in Figure 10b, it can be seen that the lateral trajectory tracking deviations of the two control strategies show significant differences. The peak lateral deviation obtained by the MPC algorithm is 1 m, while the RMPC only generates a peak deviation of approximately 0.2 m under the same working conditions. The significant increase in lateral deviation is mainly concentrated during the vehicle lane-changing process. During the attitude correction stage, this deviation gradually attenuates and approaches convergence to zero. It is worth noting that compared with MPC, the lateral deviation under the RMPC strategy has a faster stabilization characteristic, which can accelerate the approach to a steady state and achieve zero-deviation convergence.

**Figure 11:**
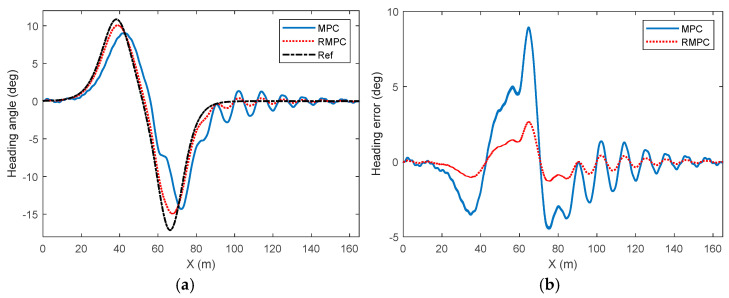
HiL test results for heading angle of vehicles under high and low speed conditions. (**a**) Heading angle, (**b**) heading error.

Figure 11 shows the HiL test results of the heading angle of the vehicle under the high-attachment low-speed condition. The influence of parameter mismatch is significantly correlated with the dynamic response characteristics of the heading angle, and its variation pattern is relatively consistent with the variation trend of the vehicle lateral deviation. As can be seen from the figure, the heading angle under the RMPC algorithm is generally in good agreement with the reference heading angle. In contrast, the heading angle under the MPC algorithm has a relatively significant lag, the curvature of the heading curve has a certain sudden change, and the overall amplitude also shows a problem of oscillation during the period when the vehicle trajectory curve returns to positive. The lag, deviation, and oscillation problems of vehicle heading angle under the MPC algorithm are also the direct reasons why the lateral deviation of vehicle driving trajectory, heading deviation, and the tracking control of vehicle yaw angle velocity are relatively far from the reference value. Similarly, in combination with the heading deviation, it can be known that in scenarios with uncertainties, the maximum value of the heading angle tracking control deviation under the MPC algorithm is close to 9 deg, while the corresponding deviation of the RMPC strategy is always constrained within 2 deg, thereby more directly reflecting the good performance of the robust model predictive control in the dynamic control of the heading angle.

**Figure 12:**
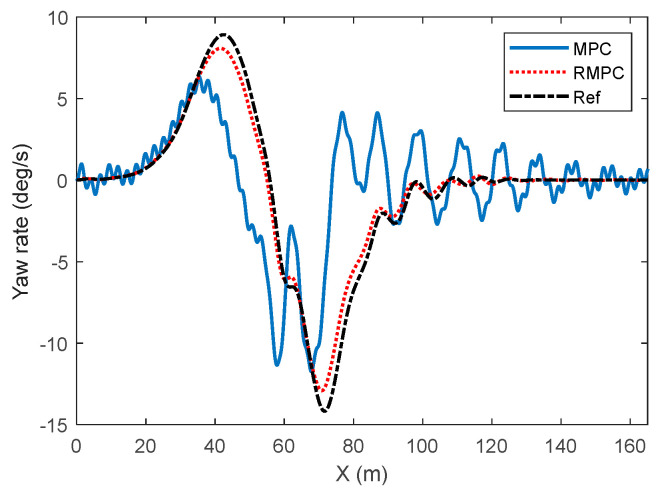
HiL test results of vehicle lateral angular velocity under high- and low-speed operating conditions.

The comparison results of the yaw velocity of vehicles under high-speed and low-speed conditions are shown in Figure 12. The simulation results show that under the influence of uncertain factors, the yaw angular velocity response under the MPC algorithm has a significant lag effect, especially presenting a tracking delay problem in the reference peak neighborhood, which is difficult to fully meet the dynamic response requirements for vehicle stability. In comparison, RMPC demonstrates superior dynamic response capability and tracking accuracy. The consistency between its actual yaw velocity and the reference trajectory has significantly improved, thereby verifying the real-time control ability of this algorithm under complex working conditions.

Under the premise of qualitative comparative analysis, the error statistics method is adopted to quantitatively characterize the vehicle control effect under the influence of uncertainty. *e_p_* is defined as the peak absolute error, *e_a_* as the average error, and *e_r_* as the root mean square error. The corresponding calculation method can be written as

(60) $$\begin{array}{l} e_{p}=x_{kpA}-x_{kpR}\\ e_{a}=\sum_{k=1}^{N}\frac{x_{kA}-x_{kR}}{N},\\ e_{r}=\sqrt{\frac{1}{N}\sum_{k=1}^{N}{\left(x_{kA}-x_{kR}\right)}^{2}} \end{array}$$

where *N* represents the total length of the discrete sampling sequence, *x_kpA_* and *x_kpR_* correspond, respectively, to the extreme values of the amplitude of the actual trajectory and the reference trajectory within the *k*-th sampling period, and *x_kA_* and *x_kR_* constitute the dynamic correspondence between the measured value and the expected value at that sampling moment. The calculation results of vehicle trajectory tracking control *e_p_*, *e_a_*, and *e_r_* under the obtained simulation conditions are shown in Table 1.

Through in-depth comparative analysis of the quantitative indicators in Table 1, it can be found that the RMPC control algorithm proposed in this study has achieved significant optimization in the core performance dimension. Compared with the traditional MPC method, RMPC shows a significant improvement in peak deviation control, effectively curbing the sharp fluctuations in the instantaneous deviations of lateral displacement and heading angle during trajectory tracking, and simultaneously enhancing the accuracy of yaw velocity tracking control, thereby significantly reducing the risk of vehicle dynamic instability caused by back-and-forth oscillations in the driving direction. Further analysis indicates that RMPC demonstrates significant advantages in both the mean error and root mean square error, two key indicators. Its global error suppression capability has been effectively verified through statistical verification, which can effectively ensure that unmanned vehicles maintain high-precision trajectory tracking capabilities under model parameter perturbation and complex road conditions. It can also achieve a stable yaw stability control function, providing a robust solution that combines tracking accuracy and driving stability for the trajectory tracking driving process of unmanned vehicles. By calculating the time consumption of single-step optimization, it can be known that under the three different working conditions of 10% parameter perturbation deviation, 30% parameter perturbation deviation, and HiL test, the solution times of traditional MPC are 3.21 s, 3.50 s, and 4.11 s, respectively. At this point, the solution times of the proposed RMPC under the same hardware and working conditions are 3.05 s, 3.43 s, and 3.65 s, respectively. The comparison results show that although RMPC increases the computational load due to robust optimization, it can still meet the control cycle requirements of 10-ms-level on-board hardware through double-layer structure compression and vertex screening.

## 6. Conclusions

This study focuses on the core issue of robust control of intelligent vehicle trajectory tracking under model uncertainty conditions, and proposes a systematic solution that integrates the convex multicellular modeling framework and the improved min-max robust model predictive control strategy. Through the precise characterization of the vehicle’s lateral–yaw coupling dynamic behavior, a multi-cell uncertain model that can explicitly represent the perturbation of tire lateral stiffness and the time-varying characteristics of longitudinal speed was constructed. On this basis, the infinite time domain robust optimization problem was transformed into a solvable form of finite-dimensional linear matrix inequalities. Theoretical analysis shows that the designed Lyapunov–Krasovskii function and the switching control invariant set mechanism not only ensure the asymptotic stability of the closed-loop system to parameter perturbations, but also effectively reduce the computational conservatism and real-time performance bottlenecks of traditional robust predictive control in high-dimensional vertex scenarios. The simulation and hardware-in-the-loop test results consistently confirm that, compared with the conventional MPC, the RMPC algorithm proposed in this paper demonstrates significantly superior control quality within the parameter perturbation range. The peak deviation of lateral displacement and the maximum error of heading angle are significantly reduced, and the tracking delay of yaw velocity is effectively suppressed. The statistical indicators *e_p_*, *e_a_*, and *e_r_* were all superior to those of the control group, verifying the algorithm’s high tolerance for model mismatch and external disturbances. In conclusion, the convex polycyte-RMPC joint framework established in this paper provides feasible and efficient theoretical and technical support for high-precision trajectory tracking and robust stable control of intelligent vehicles under complex working conditions, which has positive significance for promoting the reliable implementation of autonomous driving systems in uncertain environments.

Future research will be systematically deepened around the following four dimensions. Firstly, at the model level, it is possible to explore the possibility of combining nonlinear tire models with linear variable-parameter or affine parameter-dependent modeling techniques, which will more systematically incorporate the nonlinear characteristics of tires and time-varying parameters such as vehicle speed into the model framework. In addition, it is also possible to consider real-time estimation of key time-varying parameters and states based on nonlinear observers or machine learning methods and feed these estimated values back into the predictive model and control law to enhance the controller’s adaptability to unknown/time-varying disturbances and strong nonlinearity. Secondly, at the algorithmic level, the current online solution of RMPC relies on LMI optimization with a fixed sampling period. The next step will be to explore the event-triggered adaptive time domain strategy. By dynamically adjusting the prediction time domain and the refresh frequency of the control invariant set, computational redundancy can be reduced while ensuring robustness. Meanwhile, the parameter weight prior generated by deep learning is embedded in the initial value selection of LMI in order to achieve millisecond-level convergence in complex urban working conditions. Secondly, at the system verification level, hardware-in-loop testing has initially confirmed the feasibility of the method. Subsequently, it is planned to conduct comprehensive experiments on both closed sites and open roads based on real vehicle platforms, with a focus on evaluating the algorithm’s extreme performance in scenarios such as wet and slippery low adhesion, sudden obstacle avoidance, and V2X collaboration, and construct a fault injection test system oriented to the ISO 26262 functional safety standard [38] to verify the failure tolerance of the control loop. Finally, in the dimension of multi-vehicle collaboration, the current single-vehicle RMPC framework will be extended to a distributed model to study the coupling effect of vehicle–vehicle communication delay and topology switching on the robust feasible domain. A collaborative trajectory re-planning mechanism based on consistency constraints will be proposed to provide theoretical support for the robust operation of intelligent fleets in mixed traffic flows.

## Figures and Tables

**Table 1:** Calculation results of *e_p_*, *e_a_*, and *e_r_* in HiL test.

Error	Control Method	PRE	AVE	RMSE
Lateral offset	MPC	3.0685	0.1419	0.2111
	RMPC	0.9273	0.0498	0.0722
Heading error	MPC	12.8862	2.6808	0.9838
	RMPC	7.6397	0.0769	0.0948
Tracking error of yaw rate	MPC	8.3736	1.7961	1.6440
	RMPC	0.8216	0.1319	0.1799

## Data Availability

The authors confirm that the data supporting the findings of this study are available within the paper.
